# Acute nerve stretch and the compound motor action potential

**DOI:** 10.1186/1749-7221-6-4

**Published:** 2011-08-24

**Authors:** Mark M Stecker, Kelly Baylor, Jacob Wolfe, Matthew Stevenson

**Affiliations:** 1 Department of Neuroscience, Marshall University School of Medicine, Huntington, WV 25701 USA

## Abstract

In this paper, the acute changes in the compound motor action potential (CMAP) during mechanical stretch were studied in hamster sciatic nerve and compared to the changes that occur during compression.

In response to stretch, the nerve physically broke when a mean force of 331 gm (3.3 N) was applied while the CMAP disappeared at an average stretch force of 73 gm (0.73 N). There were 5 primary measures of the CMAP used to describe the changes during the experiment: the normalized peak to peak amplitude, the normalized area under the curve (AUC), the normalized duration, the normalized velocity and the normalized velocity corrected for the additional path length the impulses travel when the nerve is stretched. Each of these measures was shown to contain information not available in the others.

During stretch, the earliest change is a reduction in conduction velocity followed at higher stretch forces by declines in the amplitude of the CMAP. This is associated with the appearance of spontaneous EMG activity. With stretch forces < 40 gm (0.40 N), there is evidence of increased excitability since the corrected velocities increase above baseline values. In addition, there is a remarkable increase in the peak to peak amplitude of the CMAP after recovery from stretch < 40 gm, often to 20% above baseline.

Multiple means of predicting when a change in the CMAP suggests a significant stretch are discussed and it is clear that a multifactorial approach using both velocity and amplitude parameters is important. In the case of pure compression, it is only the amplitude of the CMAP that is critical in predicting which changes in the CMAP are associated with significant compression.

## Background

In a previous paper [1], the response of the compound motor action potential (CMAP) produced by peripheral nerve stimulation was studied during a pure compression injury of the nerve. Although, this is one mechanism by which a nerve might be injured during surgery, nerves can also be injured as a consequence of stretch. In order to use the CMAP as a means of warning a surgeon that a nerve is undergoing significant stretch during a surgical procedure a number of criteria must be met. First, those characteristics of the CMAP that can be measured in real time must be identified and their changes during stretch must be understood. Second, optimal means of classifying whether there is impending injury to the nerve based upon these parameters must be found. Finally, the sensitivity and specificity of these changes in predicting injury must be determined. These are the primary goals of this paper.

It is well known that stretching a peripheral nerve can cause injury. Many studies have demonstrated that stretch can damage the myelin [2-4]as well as the cytoskeleton [5,6]. The neurophysiology of stretch injury has also been investigated but primarily in regard to the subacute injury caused by limb lengthening [7-10] rather than the acute injury that may occur during a surgical procedure. In particular, the electrophysiologic characteristics of these subacute injuries may be quite different from acute injuries especially since it has been shown that longitudinal stretching of the nerve for prolonged periods is associated with a greater chance of injury at the same stretching force [11] than a brief period of stretch. Electrophysiologic studies of stretch have shown both reductions in conduction velocity and decreased CMAP amplitudes but have not evaluated the criteria that could be used to determine which electrophysiologic changes provide the first indication of acute stretch related injury.

The specific goal of this paper is to study the changes in the CMAP during acute nerve stretch and compare them to the changes seen during acute compression. In particular, conduction velocities, CMAP amplitudes, CMAP duration, and the area under the curve for the CMAP will all be studied as well as the presence of spontaneous electromyographic (EMG) activity.

## Methods

### Use of animals

Under protocol #401 approved by the Marshall University IACUC, 21 sciatic nerves from 13 normal male golden Syrian hamsters were analyzes. The data were compared with data obtained in a previous study [1] from 16 sciatic nerves from 10 normal male golden Syrian hamsters were subjected to pure compression. Of the 21 nerves in this study, 5 nerves were taken from animals sedated with pentobarbital (75 mg/kg ip) and 16 from animals sedated with isoflurane (2-3.5% titrated to maintain sedation). All hamsters were purchased from BioBreeders (Watertown, MA).

### Recording the CMAP

Recordings of the CMAP were made from the stainless steel subdermal needle electrodes (Model E2-48, Astro-Med, Inc., West Warwick,) placed in the muscles of the hind paw. The sciatic nerve was stimulated proximally at the spine using similar subdermal needle electrodes placed in tripolar fashion along the nerve with approximately 2 mm separation between the electrodes. Stimulation was accomplished with a Grass S88 stimulator connected to a Grass PSIU6 constant current isolation unit. The intensity of the stimulus was increased in the range of 2-15 mA until further increases in the stimulus intensity produced no apparent increase in the amplitude of the CMAP at the beginning of the experiment. This stimulus intensity was used throughout the remainder of the experiment. The duration of each stimulus was chosen as 0.01 msec in order to minimize stimulus artifact.

The signal from the recording electrodes was amplified by Grass Model 12 amplifiers (Astro-Med, Inc., West Warwick, RI) with the high frequency filter set at 3 kHz and the low frequency filter set at 0.3 Hz and a gain of 500. Continuous recordings of spontaneous muscle activity were amplified and directed to a loudspeaker so that spontaneous electromyographic activity could be documented as they occur in synchrony with the recorded CMAP data. The signal was digitized using a NI-USB-6259 16 bit, 1.25 MHz data acquisition module (National Instruments, Austin, TX) with a sampling rate of 30,000 Hz/channel. Stimulation was performed at a rate of 5/sec and the average of 20 traces was computed prior to saving the response. Thus, CMAP's were recorded every 4 seconds.

Each hamster's rectal temperature was monitored continuously and controlled using a warming lamp. The mean temperature for all nerves was 31°C with a standard deviation of 2.3°C. In addition, continuous recordings were made of the output of a Shimpo DFS-1 force gauge (Shimpo Instruments, Itasca, IL) with a measurement accuracy of 0.1 g. The actual force exerted on the nerve is properly measured in Newtons with the conversion being the weight measured by the force gauge divided by 102. For the sake of simplicity, the weight in grams will often be used instead of the force in Newtons in the remainder of this paper. The in-house software controlling each experiment also allowed the experimenter to make annotations that were synchronous with the CMAP recordings and enabled both manual and automatic marking of the CMAP's.

**Figure 1:**
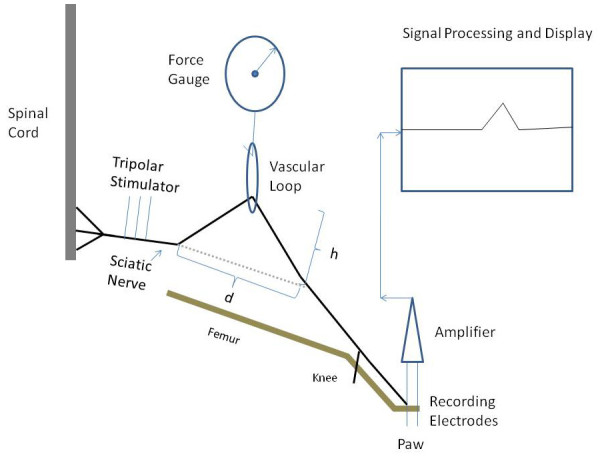
**Schematic diagram of the nerve stretch experiment**.

**Figure 2:**
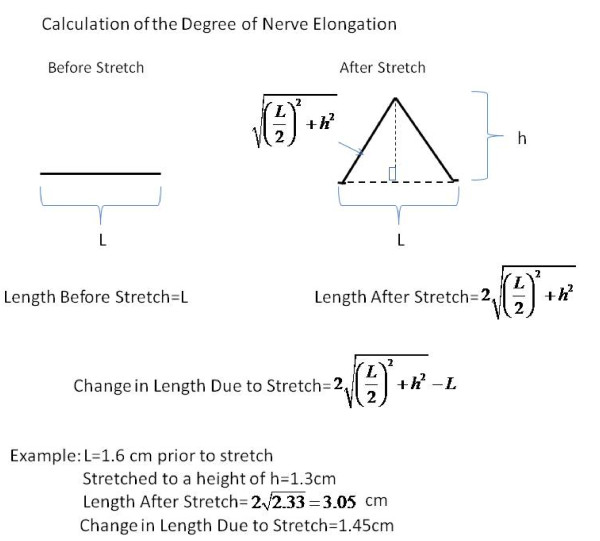
**Computation of the degree of elongation of the nerve during stretch**.

After dissection of the sciatic nerve, standard 1.3 mm wide vascular loops were wrapped around the nerve as shown in Figure 1 and then around the force gauge as the nerve was lifted out of the incision site. It should be noted that the part of the nerve subject to stretch was exposed to atmospheric oxygen throughout the experiment. Measurements were made of the height of the nerve above the incision (h in Figure 1) and the length of the open incision (L in Figure 1). It is important to be aware that this is not a model that involves pure stretch. Since the nerve is pulled away from the body, there is a component of both stretch and compression. It is also important to be aware that this stretching produces an elongation of the nerve which was estimated as $2\sqrt{{\left(\frac{L}{2}\right)}^{2}+h^{2}}-L$ (Figure 2).

Before recording data, the stimulus intensity was adjusted to obtain a supramaximal stimulus and the recording and stimulating electrodes were adjusted to obtain a high amplitude (> 500 μV) response.

**Figure 3:**
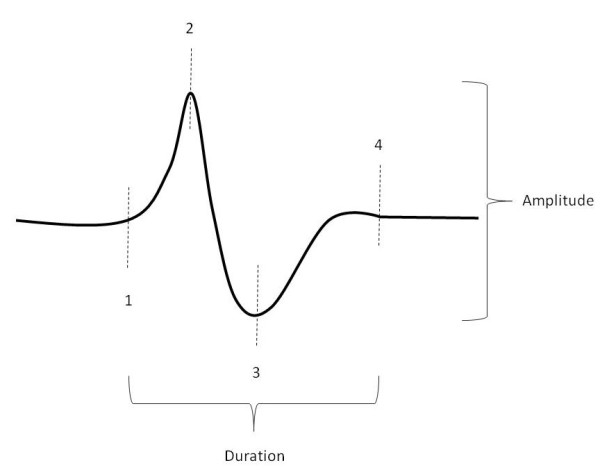
**Typical CMAP along with the points marked on that CMAP**. Note the definitions of the duration and amplitude.

Each experiment occurred in the stages noted in Table 1. Figure 3 shows a typical CMAP along with the typical points that are marked

**Table 1:** Stages of nerve stretch experiment and comparison with the nerve compression experiment

Stretch				Compression			
**Stage**	**Description**	**Maximum Force (gm)**	**Duration**	**Stage**	**Description**	**Maximum Force (gm)**	**Duration**

1	Baseline	0		1	Baseline	0	
2	First Stretch	10	3 min* Mean 3.01				
3	First Recovery	0	3 min				
4	Second Stretch	20	3 min* Mean 2.87	2	First Compression	20	3 min* Mean 3.5
5	Second Recovery	0	3 min	3	First Recovery	0	3 min
6	Third Stretch	40	3 min* Mean 1.78	4	Second Compression	80	3 min* Mean 1.78
7	Third Recovery	0	3 min	5	Second Recovery	0	3 min
8	Fourth Compression	Until 0 Amplitude	3 min* Mean 4.41	6	Third Compression	Until 0 Amplitude	3 min* Mean 1.91
9	Fourth Recovery	0	3 min	7	Third Recovery	0	3 min

This table also shows sequence of force application during an experiment. It should be noted that in stretch stages 2 4 and 6 if the CMAP amplitude fell to half of its baseline, then the stretch was immediately released. In stage 8, when the CMAP amplitude reached zero, the stretch was immediately released. Note that leg 8 is longer than the other legs because of the extended time it took to gently create the higher stretch forces.

*Planned duration. The number below this is the actual mean duration of the given stage.

### Statistical analysis

The term latency always refers to the time delay between the stimulus and the onset of the CMAP (marker 1 in Figure 3) and the term amplitude refers to the maximum peak to peak amplitude. Computation of conduction velocities assumed a synaptic delay of 0.5 msec [12]. All latencies were corrected to the values corresponding to 37°C according to the relation derived from an analysis of baseline latencies [1]:

(1) $$\text{Latenc}{\text{y}}_{\text{corrected}}=\text{Latency}*e^{-.032*\left(37-T\right)}$$

where T is the rectal temperature at the time of the latency measurement and the corrected latency is that expected at 37°C. In addition, a "corrected" velocity is also computed using instead of the linear distance from the point of stimulation to the point of recording that distance plus the amount the nerve is lengthened by the stretch.

The duration of the CMAP is measured as the difference between the time of the first and last noticeable deflection of the CMAP (the time difference between points 1 and 4 in Figure 3). Another characteristic of the CMAP is the area under the curve (AUC) Since the CMAP generally has components above and below baseline, the area under the curve is computed using Simpson's rule applied to the absolute value of the CMAP

(2) $$\text{AUC}=\overset{t_{\max}}{\underset{{t^{\prime \prime }}_{\max}}{\int }}\left|V\left(t\right)\right|dt$$

where t_start _is the shortest time after stimulation at which reliable data is available and t_stop _is the latest time (> point 4 in Figure 3) for which a CMAP is present. Because the CMAP shape and amplitude depend on the exact placement of the recording electrodes, the actual value of the measured parameters is divided by the mean value of that parameter in the baseline state (Stage 1) to arrive at "normalized" parameter values.

A number of statistical techniques are important in analyzing the data from this experiment. A Spearman rank correlation analysis (Statistica, Tulsa OK) is used to determine how independent the 5 CMAP measurements described above are. High rank correlation coefficients between two measurements would suggest that they contain similar information and are redundant descriptors of the data. In addition, a repeated measures ANOVA using the 5 measurements (MEASURE) as a repeated measure and the stage (STAGE) as an independent variable will be used to determine whether there is a statistically significant difference between the different measures in different stages. This analysis is not based upon the raw data set because this data set has many measurements for each condition and may thus produce a false statistical significance because of the large number of data points. Instead, prior to the ANOVA analysis, a reduced file is created that has the mean value of each normalized measure in each leg for each nerve. This is the file that is subjected to statistical analysis. A similar (STAGE × MEASURE × ANESTHESIA) repeated measures ANOVA is used to determine whether anesthesia has any effect on the measures and whether that effect is dependent on the degree of stretch.

From the neurophysiologic monitoring standpoint, it was important to determine the time at which the first statistically significant changes in one of the above discussed CMAP parameters occurred during the experiment. A simple method to determine this time involved performing a repeated measures ANOVA in the normalized variable under study starting with the first two stages of the experiment and then adding successive stages to the ANOVA until a statistically significant effect is noted. The reduced size file is used for this analysis.

Finally, it was important to investigate the neurophysiologic parameters that distinguished nerves subjected to different stretching forces. This was done by carrying out linear discriminant analyses (Statistica, Tulsa OK) with the dependent variable being the stage and the independent variables being all or a subset of the normalized measurements. When more than one independent variable was used a linear stepwise analysis was carried out with an F to enter of 3 and an F to remove of 1. Accuracy of the classification was recorded as were the classification functions. Multiple such analyses were carried out to compare the baseline CMAP data from that in each stage where there was nerve compression. This was carried out separately for each of these stages since the criteria for detection were likely to be different. These same analyses were carried out on the data obtained in a previous set of experiments on the changes in the CMAP during pure nerve compression [1].

## Results

### Nerve Breakage

For 16 nerves, information was available on the force at which the nerve breaks into two different segments. This occurs at a mean force of 331 gm with a standard deviation of 55 gm. In 14 nerves, the nerve broke at the distal incision, in one case the nerve broke at the proximal incision site and in 1 case, the nerve broke at the location of the vascular loops.

### Force Required to Abolish the CMAP

It should be noted that the CMAP reached zero amplitude at a mean of 73 gm force with a range of 41-120 gm and a standard deviation of 18 gm. This is roughly 22% of the force required to break the nerve.

### Changes in CMAP during Nerve Stretch

#### Independent Variables

There are a large number of potentially interesting variables describing the CMAP. Because of this, it was important to know which variables contained unique information. To achieve this, a Spearman rank correlation analysis (Table 2) is performed with all of the normalized measured variables both when the entire data set and when the data set contained only the first 7 segments of the experiment. When the total data set was used, there was significant statistical correlation between all of the normalized outcome variables at the p < 0.001 level. The strongest correlations were between the area under the curve (AUC) and the normalized amplitude (R = 0.82) and adjusted normalized velocity and normalized velocity (R = .58). The lowest correlation was between the duration ratios and the amplitude and between the amplitude measures and the velocity variables. Overall correlations are lower but still significant when only the data from the first 7 experiment phases are used. Although this analysis indicates that the normalized outcome variables are strongly correlated, the Spearman rank correlation coefficients all being less than 0.82 suggests that each of the variables contains at least some unique information.

**Table 2:** Correlations between measured variables

	Normalized Amplitude	Normalized AUC	Normalized Velocity	Normalized Corrected Velocity	Normalized Duration
Normalized Amplitude		.82 (.63)	.14 (.03)	.06 (-.06)	.21 (.11)
Normalized AUC	.82 (.63)		.20 (.15)	.13 (.07)	.24 (.19)
Normalized Velocity	.14 (.03)	.20 (.15)		.62 (.56)	.31 (.20)
Normalized Corrected Velocity	.06 (-.06)	.13 (.07)	.62 (.56)		.35 (.27)
Normalized Duration	.21 (.11)	.24(.19)	.31 (.22)	.35(.27)	

The entries in the table are Spearman rank correlation coefficients. All are significant at p < .001 using all of the stages. Using data only from stages 1-7 gives the data in parentheses.

The statistical difference between the 5 outcome measures during the stretch experiment can also be estimated using a repeated measures ANOVA with stage as the independent factor and the normalized outcome variables as 5 repeated measures. There was a significant main effect of STAGE (F(6,140) = 4.1 p < .001) and outcome variable (MEASURE) (F(4,560) = 8.7 p < .001) as well as a significant interaction term (F(24,560) = 1.75; p < .02). This again suggests that the 5 outcome measures have different dependence on the experimental stage.

#### General Trends

**Figure 4:**
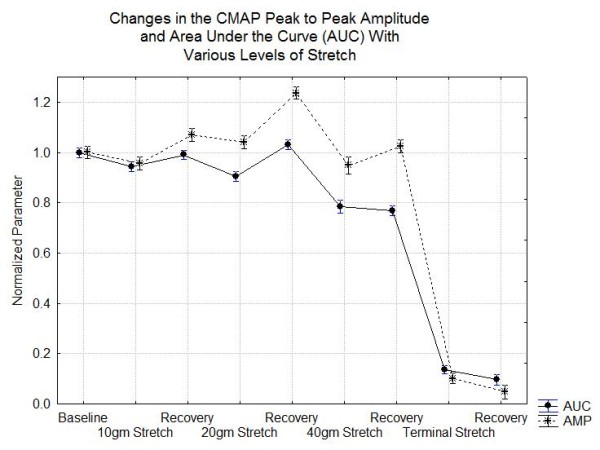
**Changes in the normalized peak to peak amplitude (AMP) and the normalized area under the curve (AUC) during the stretch experiments**.

**Figure 5:**
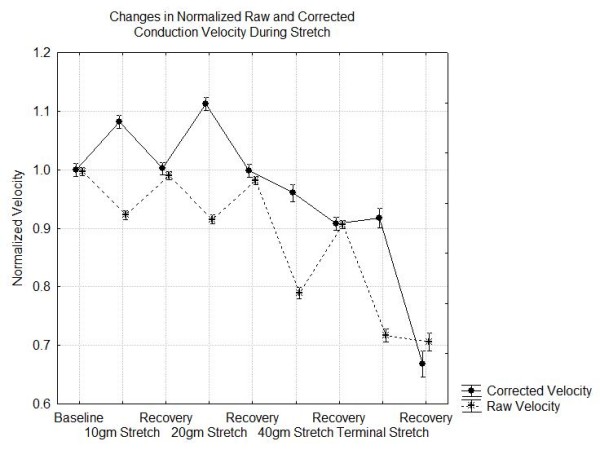
**Changes in the normalized nerve conduction velocity during various phases of the nerve stretch experiment**.

**Figure 6:**
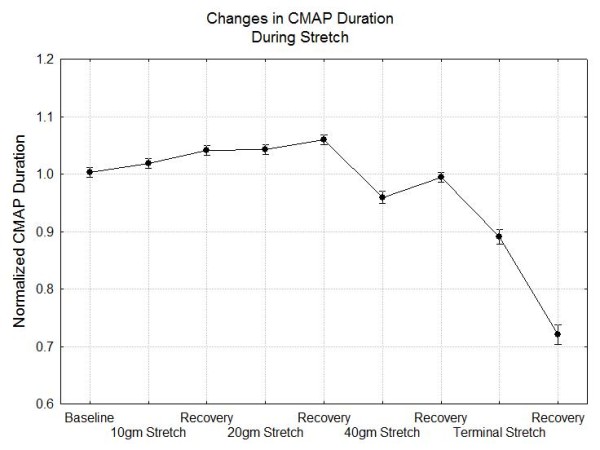
**Changes in the normalized CMAP duration during the stretch experiments**.

The overall results of the experiments are summarized in Figures 4, 5 and 6. Figure 4 shows the changes in the CMAP peak to peak amplitude and AUC during each stage of the experiment. In this figure it is evident that the AUC drops about 5% at 10 gm stretch, 10% at 20 gm stretch and 20% at 40 gm stretch while recovering to baseline after 10 and 20 gm stretch but not after stretch with 40 gm or greater. With stretch forces less than 40 gm, the peak to peak amplitudes show significant rebound with higher amplitudes during the recovery periods than baseline although each compression does produce a relative decrease in amplitude from its pre-compression baseline. Figure 5 shows that there are significant reductions in the normalized raw velocity even at the 10 gm and 20 gm stretch conditions but even with the maximal compression, as long as response is recordable, the conduction velocity is always greater than 70% of baseline. Of course, since the nerve lengthens with stretch, the length of nerve traversed by the nerve impulses increases. Correcting for this, the actual speed of nerve conduction may be increased above baseline for stretch forces less than 40 gm. However, at the 40 gm or more stretch even the corrected velocities decline. Figure 6 shows that the duration of the CMAP increases slightly at the lowest stretch tension and then declines at 40 gm and above.

#### Individual Variability

**Figure 7:**
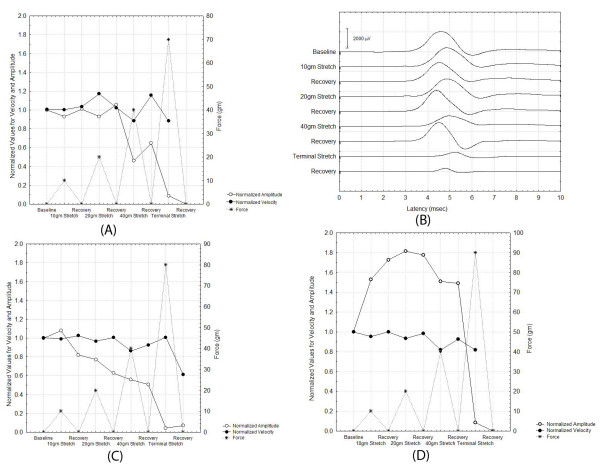
**Illustration of the differences in the responses of various nerves to stretch and the typical CMAP waveforms recorded**.

The above summary results belie the complexity of the results from individual nerves. Figure 7a shows the changes in CMAP's during a typical experiment while Figure 7b shows the actual CMAP waveforms during this experiment. Figures 7c and 7d show the dependence of the normalized peak to peak amplitude and the normalized AUC in two other nerves experiments. It is clear that the amplitude of the CMAP changes can exhibit many different patterns for stretch at < 40 gm but, for stretching forces above 40 gm, the CMAP reliably declines precipitously. The changes in velocity are more consistent from nerve to nerve than those of the CMAP amplitude or AUC, but the effects of stretch on CMAP duration also show significant variability.

In order to find the first stage for which statistically significant changes in one of the parameters describing the CMAP occurs, a sequence of one-way ANOVA's was carried out using each different parameter as the dependent variable and STAGE as the independent variable. Although the value of STAGE began at 2 for each ANOVA, the largest value of STAGE ranged from 3 to 9. In particular, the reduced data file in which only 1 data point is available for each stage is used in order to avoid the false statistical elevations that might occur as the result of multiple measurements in the same stage. Table 3 indicates that the velocity measures are much more sensitive to changes at low stretch forces than the amplitude or duration measures. In addition, the AUC ratio is more sensitive than peak to peak amplitude ratios at low stretch forces and the duration alone does not show statistically significant changes until the highest levels of stretch force.

**Table 3:** First experiment phase in which a significant change is noted in the given variable

Variable	First Stage Significant	Significance at First Significant Stage	Significance at Stage 9
Normalized Amplitude	6	.05	< .001
Normalized AUC	6	.01	< .001
Normalized Velocity	2	.001	< .001
Normalized Corrected Velocity	2	.001	< .001
Normalized Duration	8	.002	< .001

#### Anesthesia Effects

One important question is whether the variability seen in individual stretch experiments is related to the anesthesia used. In order to see if this were true, a MEASURE × STAGE × ANESTHESIA 5 × 9 × 2 repeated measures ANOVA was performed. There were significant main effects of STAGE (F(8,154) = 17, p < .001), ANESTHESIA (F(1.154) = 4.8, p = .03) and MEASURE (F(4,616) = 27, p < .001). There was a significant effect of anesthesia on MEASURE (p < .001) but no significant triple interaction of MEASURExSTAGExANESTHESIA. In fact, the velocities and durations are similar with both anesthesia types but the peak to peak amplitude and AUC were significantly lower with pentobarbital anesthesia. The sequential ANOVA analysis described above was repeated on only the group of nerves from which data was collected under isoflurane anesthesia and statistically significant changes were not found at earlier points in the experiment.

#### Predictability

Clinically, it is important to know what changes in the CMAP predict injury to the nerve and to know the sensitivity and specificity of these predictions. In order to answer these questions, multiple linear discriminant analyses were used with all or specific subsets of the four outcome variables that would be available in real time (normalized peak to peak amplitude, normalized AUC, normalized velocity, and normalized duration) to classify CMAPs as either from baseline or from one of the compression stages (2, 4, 6 or 8). As seen in Table 4, discriminating between baseline and any of the compression states can be done with 85-95% accuracy. The specificity and sensitivity of the classifier for stage 8 versus stage 1 is 100% and 84% respectively. When a low stretch force is applied, the normalized velocity is the primary contributor to the classification function and better as a univariable predictor than any of the amplitude related variables. With the larger stretch forces (> 40 gm), the normalized peak to peak amplitude or AUC are better univariable classifiers than the velocity. The duration used alone cannot provide as good a classification as the other outcome variables.

**Table 4:** Various linear models to predict stretch injury from the outcome variables

Comparison Stages	Normalized Peak-Peak Amplitude	Normalized AUC	Normalized Velocity	Normalized Duration	Best Classification	Classifier For Compression Stage
**1-2**	Yes	Yes	Yes	Yes	87% (96,77)	VEL-0.33DUR < 0.62
	Yes	Yes	No	No	63% (81,46)	AUC < 0.94
	Yes	No	No	No	63% (77,50)	AMP < 0.94
	No	Yes	No	No	64% (95,75)	AUC < 0.95
	No	No	Yes	No	85% (82,46)	VEL < 0.96
	No	No	No	Yes	63% (76,49)	DUR > 1.02
**1-4**	Yes	Yes	Yes	Yes	84% (96,71)	-0.25AMP+VEL +0.45AUC-0.75DUR < 0.35
	Yes	Yes	No	No	67% (85,49)	-0.65AMP+AUC < 0.26
	Yes	No	No	No	67% (98,32)	AMP > 1.2
	No	Yes	No	No	65% (97,70)	AUC < .90
	No	No	Yes	No	84% (92,37)	VEL < .95
	No	No	No	Yes	72% (88,54)	DUR > 1.04
**1-6**	Yes	Yes	Yes	Yes	93% (100,81)	-0.074AMP+VEL+ 0.24AUC+0.23DUR < 0.97
	Yes	Yes	No	No	79% (97,48)	-0.33AMP+AUC < 0.52
	Yes	No	No	No	63% (100,0)	----
	No	Yes	No	No	65% (100,82)	AUC < .74
	No	No	Yes	No	93% (98,31)	VEL < .85
	No	No	No	Yes	70% (99,17)	DUR < .86
**1-8**	Yes	Yes	Yes	Yes	96% (100,84)	0.48AMP+VEL+ 0.74AUC-0.98*DUR < 0.68
	Yes	Yes	No	No	96% (100,93)	0.66AMP+AUC < 0.95
	Yes	No	No	No	96% (100,93)	AMP < .30
	No	Yes	No	No	95% (100,61)	AUC < .59
	No	No	Yes	No	89% (99.8,92)	VEL < .75
	No	No	No	Yes	81% (100,32)	DUR < .82

VEL is the normalized velocity, AMP is the normalized peak to peak amplitude, DUR is the normalized duration and AUC is the normalized area under the curve. Under best classification the top number is the total number of correctly classified cases. The two numbers in parentheses below this are the specificity and sensitivity.

**Figure 8:**
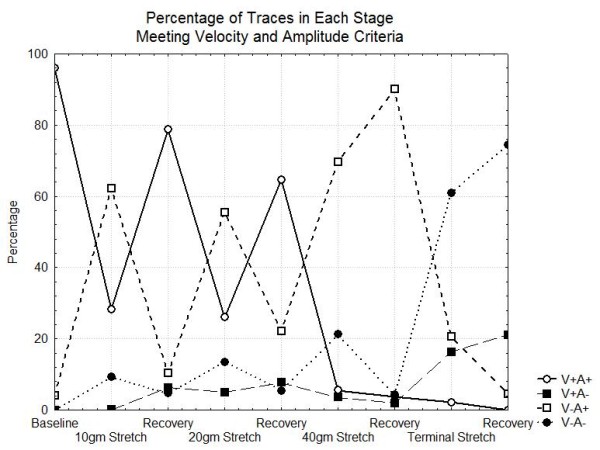
**Fraction of traces in each stage fitting the amplitude and voltage criteria or both**. V+ means normalized velocity > 0.95, V-means normalized velocity < = 0.95, A+ indicates peak to peak amplitude > 0.57, A-means peak to peak amplitude < .57.

Using multiple different criteria to classify the CMAP is important in clinical neurophysiology. Figure 8 is a graphical representation of the percentage of the traces in each stage that have normal velocities and amplitudes using the univariable classifiers developed by the linear discriminant analysis (normalized velocity abnormal if < 0.95 and normalized peak to peak amplitude < 0.57). This figure shows that the probability that both velocity and amplitude are normal (V+A+) is very low for stretch > 40 gm. The number where both are abnormal (V-A-) becomes high only when during the terminal stretch stage.

For comparison, the same analysis is carried out with the compression data from the previous paper [1]. These results are summarized in Table 5. This table demonstrates that, for nerve compression, amplitude is a better predictor of compression induced changes than velocity even at low compressive forces, although the predictability increases with higher compression forces.

**Table 5:** Various linear models to predict compression injury from the outcome variables

Comparison Stages	Normalized Peak-Peak Amplitude	Normalized AUC	Normalized Velocity	Duration	Best Classification	Classifier
**1-2**	Yes	Yes	Yes	Yes	63% (58,66)	0.17AMP+VEL -0.12AUC -0.18DUR < .86
	Yes	No	No	No	54% (29,75)	AMP < 1.05
	No	Yes	No	No	49% (5,87)	AUC < .88
	No	No	Yes	No	49% (39,72)	VEL < 1.0
	No	No	No	Yes	62% (34,57)	DUR > .98
**1-4**	Yes	Yes	Yes	Yes	86% (92,77)	0.61AMP+VEL +0.55AUC < 1.81
	Yes	No	No	No	86% (99,65)	AMP < .69
	No	Yes	No	No	81% (96,57)	AUC < .74
	No	No	Yes	No	76% (98,36)	VEL < .93
	No	No	No	Yes	52% (34,67)	DUR > 1.29
**1-6**	Yes	Yes	Yes	Yes	97% (99.7,91)	AUC-0.12DUR < 0.48
	Yes	No	No	No	95% (99,89)	AMP < .57
	No	Yes	No	No	96% (95,75)	AUC < .58
	No	No	Yes	No	85% (100,62)	VEL < .89
	No	No	No	Yes	82% (95,55)	DUR < .58

Under best classification the top number is the total number of correctly classified cases. The two numbers in parentheses below this are the specificity and sensitivity.

#### Spontaneous EMG Activity

**Figure 9:**
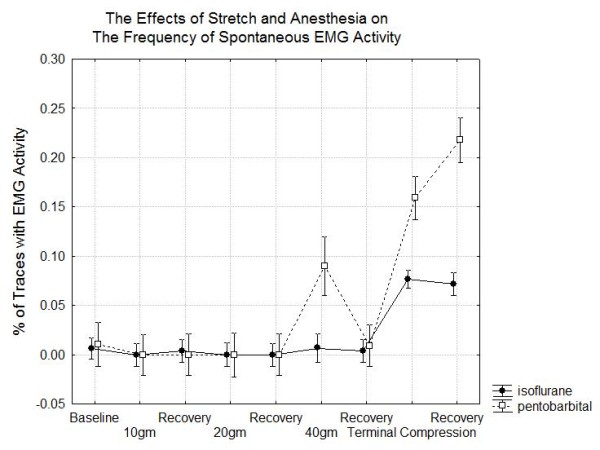
**Changes in spontaneous electromyographic (EMG) activity during the experiment**.

Clinically, the presence of spontaneous EMG activity is one of the factors used in determining when there is a significant injury to a nerve. In order to understand how the presence of spontaneous EMG activity depends on the stretching force, the CMAP and anesthesia, a factorial ANOVA is performed with EMG activity as the dependent variable and ANESTHESIA and STAGE as independent factors. In this analysis there were significant main effects of STAGE (F(8,171) = 6.4, p < .001) but not ANESTHESIA (F(1,171) = 3.2, p < .08) and there was no significant interaction (F(8,171) = .82, p < .58). This is consistent with the observations of Figure 9 that the presence of EMG activity mainly occurred during stretch at the higher force levels and during recovery after a severe stretch injury. As in the previous paper [1], EMG activity was more likely when the CMAP amplitude was significantly reduced from baseline. In particular, the value of the normalized peak to peak amplitude was 0.14 when EMG activity was heard and 0.80 when no EMG activity was heard (t = 17.5 df = 8624 p < .001). Similarly EMG activity was significantly associated with reduced normalized velocities (0.85 when spontaneous EMG present and 0.92 when such activity was not present p < .001) and reduced duration ratios (0.93 when EMG present and 1.0 when EMG absent p < .001).

#### Does the Effect of Low Stretch Levels Predict the Response to High Stretch Levels?

Since this experiment involves multiple sequential stretches of a nerve, it is useful to ask whether the response to a low level of stretch predicts the response to a higher level of stretch. As a partial answer to this question, multiple Spearman rank correlation analyses were performed between the value of the outcome variables in one stage and other stages. Because of the large number of comparisons involved, a Bonferroni correction was made and significance tested at the .001 level. The results are shown in Table 6. There was a strong positive correlation (R = 0.8 p < .001) between the minimum velocity in stage 2 and the minimum velocity in stage 4 but not stage 6. Similarly, there was a positive correlation (R = .85, p < .001) between the minimum AUC in stage 2 and stage 4 although a similar relation was not seen for the peak to peak amplitudes. There was also a positive correlation between the duration in stages 2 and 4

**Table 6:** Significant correlations in outcome variables (minimum normalized amplitude, minimum AUC, minimum normalized velocity, minimum duration) in different stages

	Stage 2	Stage 4	Stage 6	Stage 8
Stage 2	--	(VEL,VEL)	N.S.	N.S.
		(AUC,AUC)		
		(DUR,DUR)		
Stage 4		--	N.S.	N.S.
Stage 6			--	(VEL, VEL)
				(VEL, DUR)
Stage 8				--

Statistical significance level set at .001 because of multiple testing.

## Discussion

From a clinical standpoint, it is critical to understand how different types and severity of nerve injury affect the CMAP so that the CMAP can be used to predict when there is significant injury to a nerve. Many criteria have been used to interpret intra-operative neurophysiologic studies [13] and these depend on the specifics of the surgical procedure, the structures at risk and the specific testing modality [14-18]. Despite this, the most commonly used criteria for deciding when there is a significant change in somatosensory evoked potentials is either a 10% reduction in velocity (or 10% increase in latency) or a 50% reduction in amplitude. For transcranial motor evoked potentials the criteria are often taken as complete disappearance of the potential rather than a 50% decrease in amplitude.

One difficulty with clinical studies to assess the best warning criteria is that it is often impossible to know the exact timing and magnitude of the forces applied to a monitored nerve during a surgical procedure. The other difficulty is that the clinical outcome of the surgical procedure is not known until the procedure is over. Thus, if the surgeon is provided a warning based upon the one set of criteria and corrective action is taken, it is impossible to decide whether the criteria used to provide the warning yielded a false positive warning or accurately identified a true impending injury to the nerve that was corrected. Hence, experimental studies on animals can provide useful complementary information. In studies of stretch related to limb lengthening, Jou [19] suggests that a 50% change in a somatosensory evoked potential amplitude is associated with a clinical deficit due to stretching of the peripheral nerve. Wall [9] found that stretching a nerve to a strain of 6% longitudinally in rabbit tibial nerve produced a 70% reduction in the nerve action potential and at 12% strain conduction was blocked and never recovered fully. In the current study, strain was not longitudinal (in fact it was primarily perpendicular to the axis of the nerve) as in other studies but had a magnitude up to 35%. The result of Wall were confirmed by studies of Brown [8] on the CMAP showing that 15% strain produced a 99% reduction in amplitude and Li [10] showing severe conduction block in nerve action potential at strains of 20%. The current study did not include outcome measures but the study of Fowler [11] in rat sciatic nerve indicated that those nerves could tolerate 50 gm of stretch for 2 minutes before permanent injury ensued. The hamster sciatic nerve is much smaller than the rat and is likely more susceptible to injury. This provides evidence that the highest stretch levels used in this study would likely have been associated with a clinical deficit in a survival study.

In terms of interpretation criteria, for stretch forces < 40 gm, the main effect is an increase in latency and decrease in the standard velocity measure during nerve stretch, with velocity changes as low as 5% being significant At stretch forces > 40 gm, the changes in amplitude and area under the curve are more significant and better able to classify the changes in the CMAP than the velocity. This is different from the case of a purely compressive injury where the amplitude of the CMAP is always the best variable for classifying signals as being from baseline or one of the compression stages even at low compression force. This is the expected result since in the pure localized compression model, conduction abnormalities develop in a segment of the nerve that is small in comparison to the distance between the stimulating and recording electrodes. Thus, even if there were a severe reduction in conduction velocity in this small length, the overall conduction velocity would change little. In this particular model, at low stretch forces, the degree of compression at the point where the vascular loop transfers force to the nerve is too small to cause conduction block and so the amplitude does not decrease significantly. However at high stretch forces, there is significant compression at the point where the vascular loops transfer force to the nerve and the amplitude declines. For the low stretch forces, the increase in conduction velocity is unlikely to be related to a change in the passive properties of the axon since the diameter of the axon must decline as its length increases in order to maintain a constant volume and axons with smaller diameters have reduced conduction velocities. It also cannot be related to a change in the distribution of conducting axons since the conduction velocity is computed from the onset latency and so reflects the velocity of only the most rapidly conducting axons. Also, because of the very short stimulus durations used, only the largest and most rapidly conducting axons are tested in this paradigm. The most probable explanation is that stretch affects some of the properties of ion channels and hence excitability of the axonal membrane [20-23]. This might also be a likely explanation for the fact that the CMAP amplitude often becomes larger after mild degrees of compression than at baseline especially if the small degree of stretch depolarizes the membrane slightly and increases excitability. An analogous phenomenon is seen after an axon is exposed to low doses of 4-aminopyridine which at low doses blocks potassium channels and increases excitability but at high doses reduces excitability [24,25]. However, in order to verify this hypothesis, additional experiments studying the membrane properties of the stretched axons would be needed.

Returning to the clinical question regarding CMAP based decision criteria, it is clear that is important to look at many different characteristics of the CMAP. Even 5% reductions in the conduction velocity can signal that a nerve has been subjected to a significant stretch. Although changes of this magnitude in only the velocity are associated with good recovery after the stretch is released, they still would provide a valuable early warning to a surgeon. The peak to peak amplitude is more variable during the nerve stretch experiments but both velocity and amplitude are abnormal when there is a high level of stretch. However, a high level of sensitivity of the CMAP for predicting high stretch levels cannot be achieved unless as demonstrated in Figure 9, an abnormality in either velocity or amplitude is considered significant. The presence of both increases specificity.

It is important to be cautious in generalizing this information to human recordings for at least 2 reasons. First, in most clinical situations, the nerve is not exposed to atmospheric oxygen as in this experiment and so is much more sensitive to the effects of change in blood flow [26] than the nerves in this experiment. Second, the composition of and the amount of connective tissue are different in human and hamster nerves [27]. Despite these limitation, there are some possible clinical implications that may be helpful for intra-operative neurophysiologic monitoring. First, spontaneous EMG activity may not be the first sign of injury to a nerve and its presence or absence may be strongly influenced by anesthesia. Second, the type of change to be expected in the CMAP depends on the mechanism of injury. Early changes in the velocity occur with stretch while with compression over small areas, the first changes are in amplitude. However, when there is significant injury, there is a decline in amplitude no matter what the mechanism.

## Competing interests

The authors declare that they have no competing interests.

## Authors' contributions

MS participated in study design, data collection, data analysis, and writing of the paper. KB and MS participated in data collection, data analysis and in writing of the paper. JW participated in the data analysis and the data collection. All authors have read and approved the final version of the manuscript.
